# Nanomaterials in biomedicine, drug delivery and pharmaceutical analysis

**DOI:** 10.5599/admet.2125

**Published:** 2023-10-16

**Authors:** Hassan Karimi-Maleh, Afsaneh L. Sanati, Rozhin Darabi

**Affiliations:** 1 School of Resources and Environment, University of Electronic Science and Technology of China, P.O. Box 611731, Xiyuan Ave, Chengdu, PR China; 2 School of Engineering, Lebanese American University, Byblos, Lebanon; 3 Institute of Systems and Robotics, Department of Electrical and Computer Engineering, University of Coimbra, Polo II, 3030-290, Coimbra, Portugal

After the introduction of nanotechnology as a new science, there were tremendous changes in its application. Nanomaterials rapidly penetrate a diverse area of biomedicine and pharmaceutical research, including drug development, drug delivery, tissue engineering and medicinal chemistry. Its versatile use helps in alleviating complex difficulties faced with drug administration and absorption, controlled release, targeted delivery and cellular uptake. Due to their distinctive physico-chemical properties and ability to form various solid-state formulations, they find increased use in the preparation of new drug formulations, imaging techniques and particularly in medicinal chemistry as catalysts for the sensitive determination of drugs and other biologically active compounds. The role of nanomaterials as catalysts in the preparation of substances with pharmacological properties, the preparation of sensors, or the degradation and removal of medicinal compounds polluting the environment is undeniable.

This special issue aims to cover recent advances in the preparation and application of nanomaterials in the above-mentioned fields. Its scope includes the role of nanomaterials in drug delivery and the development of new drug formulations, novel polymorphs and other solid-state forms, the influence of nanotechnology on the physico-chemical properties of importance to adsorption, distribution and metabolism of drugs, tissue and cellular uptake of nanomaterials and their treatment potential and application of nanomaterials as catalysts for the sensitive and selective determination of biologically active compounds.

The special issue is guest-edited by Hassan Karimi-Maleh, Afsaneh L. Sanati, Rozhin Darabi. The whole issue will be split into two consecutive issues. This issue, the first of the series, consists of 5 review articles and 6 original papers. Two reviews address the synthesis of nanomaterials and their applications (“Eco-friendly synthesis of chitosan and its medical application: from chitin extraction to nanoparticle preparation” and “Synthetic routes to theranostic applications of carbon-based quantum dots”). A review by D. Pylypenko (“Liposomes: from August Wassermann to vaccines against COVID-19”) covers liposomal antigens as antigen-delivery systems for diagnosis and immunoprophylaxis. Two additional reviews tackled the use of nanomaterials as therapeutics (“Supremacy of nanoparticles in the therapy of chronic myelogenous leukemia” and “Infections associated with SARS-CoV-2 exploited via nanoformulated photodynamic therapy”). The original papers present the preparation and application of nanoparticles of different materials such as cyclodextrin, silica, selenium, silver, chitosan and polyvinyl alcohol.

